# Very long term outcome after linear versus electrogram guided ablation for persistent atrial fibrillation

**DOI:** 10.1038/s41598-021-02935-3

**Published:** 2021-12-08

**Authors:** Seigo Yamashita, Michifumi Tokuda, Saagar Mahida, Hidenori Sato, Hirotsugu Ikewaki, Hirotsuna Oseto, Masaaki Yokoyama, Ryota Isogai, Kenichi Tokutake, Kenichi Yokoyama, Ryohsuke Narui, Mika Kato, Shin-ichi Tanigawa, Ken-ichi Sugimoto, Michihiro Yoshimura, Teiichi Yamane

**Affiliations:** 1 Division of Cardiology, Department of Internal Medicine, The Jikei University School of Medicine, 3-19-18 Nishishinbashi, Minato-ku, Tokyo, Japan; 2 Department of Cardiac Electrophysiology, Liverpool Heart and Chest Hospital, Liverpool, UK

**Keywords:** Cardiology, Medical research

## Abstract

The optimal ablation strategy for persistent atrial fibrillation (PsAF) remains to be defined. We sought to compare very long-term outcomes between linear ablation and electrogram (EGM)-guided ablation for PsAF. In a retrospective analysis, long-term arrhythmia-free survival compared between two propensity-score matched cohorts, one with pulmonary vein isolation (PVI) and linear ablation including roof/mitral isthmus line (LINE-group, n = 52) and one with PVI and EGM-guided ablation (EGM-group; n = 52). Overall, 99% of patients underwent successful PVI. Complete block following linear ablation was achieved for 94% of roof lines and 81% of mitral lines (both lines blocked in 75%). AF termination by EGM-guided ablation was accomplished in 40% of patients. Non-PV foci were targeted in 7 (13%) in the LINE-group and 5 (10%) patients in the EGM-group (p = 0.76). During 100 ± 28 months of follow-up, linear ablation was associated with superior arrhythmia-free survival after the initial and last procedure (1.8 ± 0.9 procedures) compared with EGM-group (Logrank test: p = 0.0001 and p = 0.045, respectively). In multivariable analysis, longer AF duration and EGM-guided ablation remained as independent predictors of atrial arrhythmia recurrence. Linear ablation might be a more effective complementary technique to PVI than EGM-guided ablation for PsAF ablation.

## Introduction

While pulmonary vein isolation (PVI) is associated with high success rates in patients with paroxysmal atrial fibrillation (PAF)^1–4^, ablation outcomes in patients with persistent AF (PsAF) are more modest. In patients with PsAF, additional substrate-based AF ablation strategies have demonstrated potential benefit in a previous meta-analysis of controlled studies^5^. Linear ablation including roof and mitral isthmus (MI) lines^6^ and electrogram (EGM)-guided ablation^7,8^ techniques have been widely used in this context. However, the reported outcomes following ablation with these techniques have been variable^9,10^. In a previous multicenter randomized trial, additional AF substrate ablation (roof and MI lines or complex fractionated atrial electrogram [CFAE] or EGM-guided) beyond PVI for PsAF was not associated with incremental benefit compared to PVI alone. Importantly, while AF/atrial tachycardia (AT)-free survival rates were relatively low (around 40%), operator bias for linear ablation and CFAE-guided ablation should be considered^11^. There is a paucity of data on the relative efficacy of linear ablation and CFAE or EGM-guided ablation for PsAF^11,12^. In the present study we compared very long-term clinical outcomes of linear ablation and EGM-guided ablation.

## Results

### Patient characteristics

The overall cohort consisted of 104 patients (mean age 53 ± 11 years; 2 [1.9%] female). The average minimum continuous AF duration was 15 ± 20 months. 29 (28%) had PsAF lasting between 6 months and 1 year while 43 (41%) had PsAF for > 1 year. Mean left atrial diameter (LAD) and left ventricular ejection fraction (LVEF) were 42 ± 4.9 mm and 62 ± 7.5%, respectively. The number of failed anti-arrhythmic drugs (AADs) before catheter ablation (CA) was 1.4 ± 1.0 (including 65 [78%] on class III AAD; Bepridil [150–200 mg] and 3 [2.9%] on amiodarone). Beta-blockers were used in 39% of patients. Patient characteristics after propensity score-matched analysis (final cohort of 52 patients in the LINE-group and EGM-group) are shown in Table 1.

**Table 1:** Baseline patients’ characteristics.

	Total (n = 104)	LINE-group (n = 52)	EGM-group (n = 52)	p-value
Age, years old	53 ± 11	52 ± 10	54 ± 12	0.49
Female, n (%)	2 (1.9%)	2 (4%)	0 (0%)	0.50
Minimum continuous AF duration, month	15 ± 20	15 ± 18	16 ± 21	0.80
Long-standing PsAF (> 1 year), n	43 (41%)	22 (42%)	21 (40%)	1.00
Unknown time of AF onset, n	78 (75%)	42 (81%)	36 (69%)	0.26
BMI, kg/m^2^	25 ± 3.7	26 ± 4.2	25 ± 3.3	0.35
Number of failed AADs, n	1.4 ± 1.0	1.3 ± 1.0	1.5 ± 1.0	0.50
Structure heart disease, n	8 (8%)	5 (10%)	3 (6%)	0.72
CHA2DS2-VASc score, n	0.8 ± 0.9	0.8 ± 0.9	0.8 ± 0.9	1.00
Hypertension, n	53 (51%)	27 (52%)	26 (50%)	1.00
Diabetes Mellitus, n	10 (10%)	5 (10%)	5 (10%)	1.00
LAD, mm	42 ± 4.9	42 ± 5.1	41 ± 4.7	0.31
LVEF, %	62 ± 7.5	62 ± 8.2	63 ± 7.0	0.55
BNP, pg/ml	85 ± 61	82 ± 57	91 ± 65	0.49
Ccr, mg/dl	80 ± 17	81 ± 17	78 ± 18	0.41

*AAD* anti-arrhythmic drugs, *AF* atrial fibrillation, *BMI* Body mass index, *BNP* brain natriuretic peptide, *Ccr* creatinine clearance, *LAD* left atrial diameter, *LVEF* left ventricular ejection fraction.

### Acute procedural outcomes

As shown Table 2, 12 (23%) patients in LINE-group and 5 (10%) patients in EGM-group were in sinus rhythm (SR) at the beginning of the procedure. In the EGM-group, AF was induced by burst pacing in all patients with SR at the beginning of the procedure. PVI was performed as a first step in all patients, with a 99% successful isolation rate. Electrical cardioversion was performed either before PVI, after PVI or after linear ablation. In the EGM-group AF persisted post-PVI in all but 1 patient.

**Table 2:** Ablation results in the initial CA.

	LINE-group (n = 52)	EGM-group (n = 52)	p-value
Start in AF rhythm, n	40 (77%)	47 (90%)	0.11
Successful PVI (No. of PVs)	204/206 (99%)	203/205 (99%)	1.00
Successful roof line, n	49/52 (94%)	2/3 (67%)	–
Successful MI line, n	42/52 (81%)	4/4 (100%)	–
AF termination by EGM-guide ablation, n	–	21 (40%)	–
SVC isolation, n	2 (4%)	1 (2%)	1.00
Non-PV foci except for SVC, n	6 (12%)	4 (8%)	0.74
Procedure time, min	252 ± 65	291 ± 56	**0.002**
Fluoroscopic time, min	137 ± 41	141 ± 32	0.64
Total RF time, min	65 ± 27	91 ± 34	** < 0.001**
RF time for PVI, min	32 ± 25	36 ± 15	0.38
Major complications, n	2 (3.8%)	1 (1.9%)	1.00
Very early recurrence of AF (< 3 days), n	16 (31%)	23 (44%)	0.22

Significant values are in bold.

*AF* atrial fibrillation, *MI* mitral isthmus, *PVI* pulmonary vein isolation, *RF* radiofrequency, *SVC* superior vena cava.

In LINE-group, roof and MI lines were successfully blocked in 49 (94%) and 42 (81%) patients, respectively. Both lines were completed in 39 (75%) patients. Epicardial ablation within the coronary sinus (CS) for the MI line was added in 28 (54%) patients. Mean radiofrequency (RF) time for roof and MI line was 6.6 ± 3.5 and 14.0 ± 7.3 min, respectively. Linear ablation was performed during AF in 25/52 (48%) patients. AF terminated to SR or common atrial flutter during linear ablation in 5 of these 25 (20%) patients.

In the EGM-group, AF terminated by RF in 21 (40%) patients (conversion to AT in 11 patients and termination to SR in 10 patients). Of the 11 patients with conversion to AT, a diagnosis was possible in 6 ATs (perimitral AT [n = 2]; roof dependent [n = 1]; common AFL [n = 2]; focal AT from MI [n = 1]). Roof and/or MI lines were eventually created in 5 patients due to induced roof and/or MI line-dependent ATs. Mean RF time for EGM-guide ablation was 47 ± 30 min. The right atrium was targeted in 36 (69%) patients.

Non-PV foci ablation, including superior vena cava (SVC) isolation, was performed in 7 (13%) in the LINE-group and 5 (10%) patients in the EGM-group (p = 0.76). Procedure time and total RF time was significantly longer in EGM-group (LINE-group vs. ECG-group; procedure time 252 ± 65 vs. 291 ± 56 min, p = 0.002; RF time 65 ± 27 vs. 91 ± 34 min, p < 0.001). There were no differences in the incidence of procedure-related major complications between the two groups (LINE-group: pericardial effusion [n = 1]; pericardial drainage [n = 1]; EGM-group: pericardial drainage [n = 1]). Very early recurrence rates (within 3 days) were also comparable between two groups (Table 2).

### Long-term outcome

**Figure 1:**
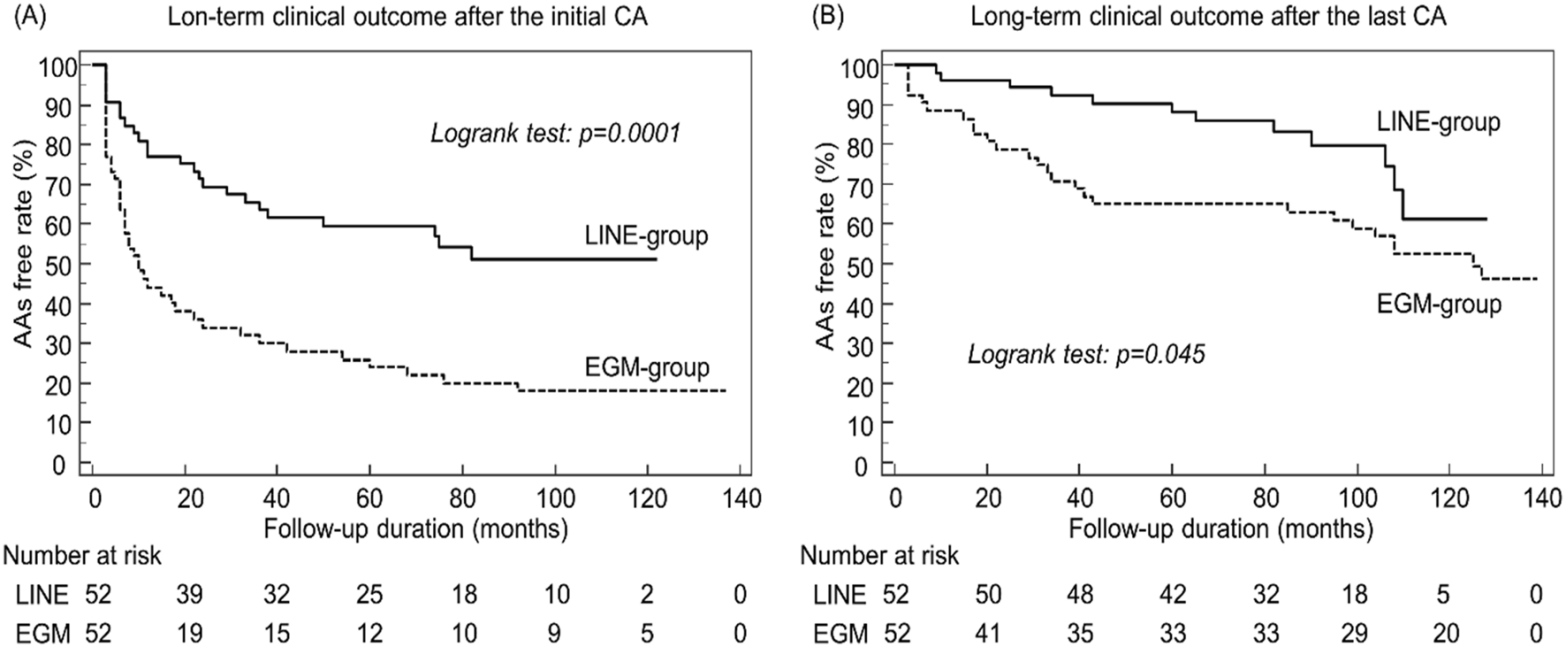
Kaplan–Meier curves of long-term clinical outcome after the initial (**A**) and last (**B**) CA procedure between LINE-group and EGM-group.

**Figure 2:**
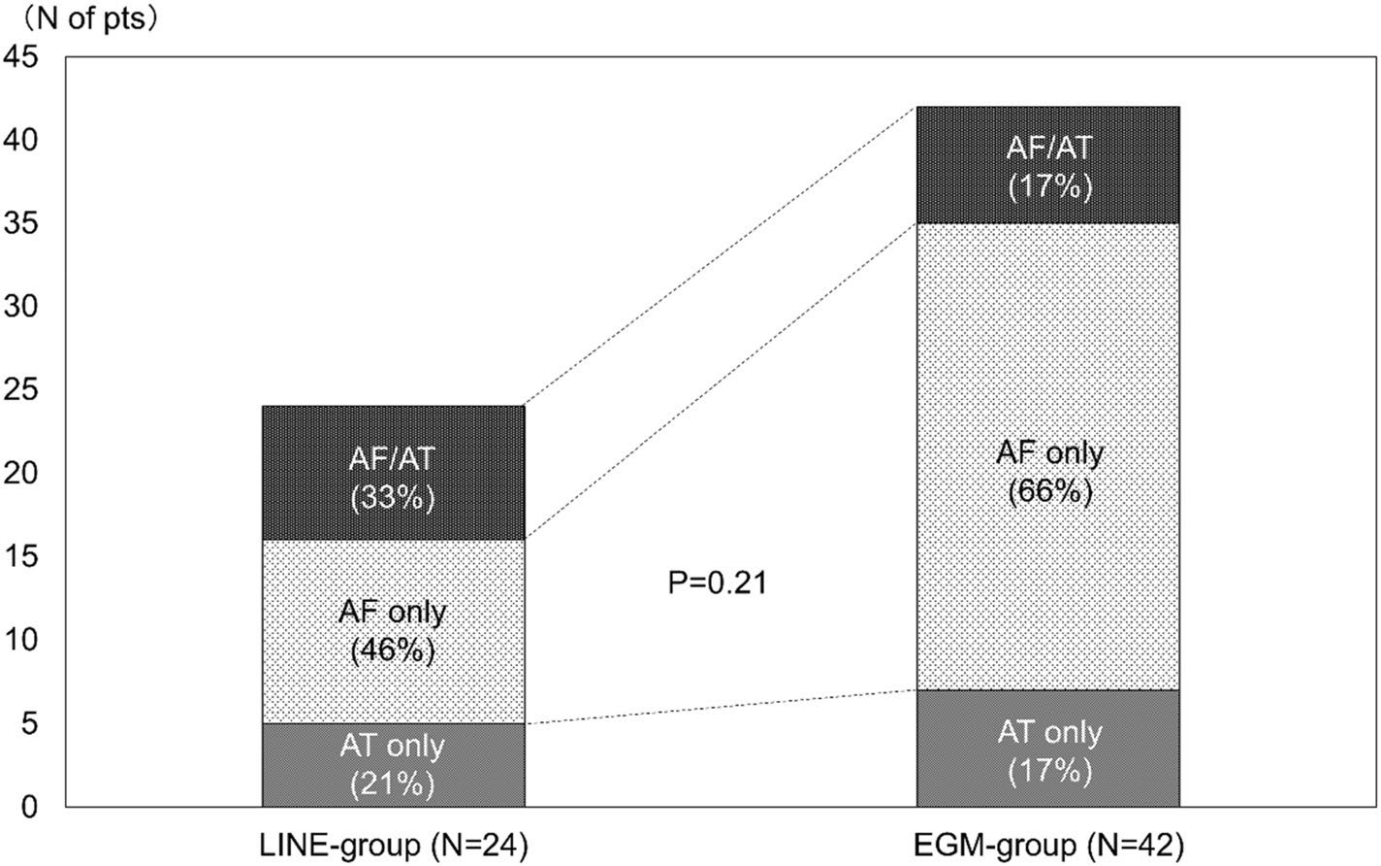
Recurrent forms after the initial CA between LINE-group and EGM-group.

During 100 ± 28 months of follow-up, the LINE-group had superior atrial arrhythmias (AAs)-free survival after the initial CA compared to the EGM-group (Logrank test: p = 0.0001, Fig. 1A). SR maintenance was consistently higher in the LINE-group compared to the EGM-group after the initial procedure (LINE-group vs. ECG-group; year 1: 79% vs. 42%; year 3: 63% vs. 31%; year 5: 60% vs. 25%; year 8: 54% vs. 19%). The majority of patients (71%) with AAs recurrence were on AAD therapy at the time of recurrence. Overall, 38/104 (37%) patients were free of AAs at 8 years after the initial CA (LINE-group [n = 28]; EGM-group [n = 10]). The proportion of AAs-free patients on AAD therapy were comparable between the two groups (LINE-group vs EGM-group; 5/28 [18%] vs 0/10 [0%], p = 0.30). AAs recurrence subtypes were similar between the LINE-group vs. EGM-groups (LINE-group vs. ECG-group; AF-only recurrence: 11 [46%] vs. 28 [66%]; AT-only: 5 [21%] vs. 7 [17%]; AF and AT 8 [33%] vs. 7 [17%], p = 0.21 Fig. 2). In addition, the pattern of AF recurrence was comparable between the two groups (LINE-group vs. ECG-group; PsAF recurrence 8 [42%] vs. 15 [44%], p = 1.00).

Among 24 (46%) and 42 (81%) patients of AAs recurrence in LINE and EGM-group, 24 (100%) and 34 (81%) of patients underwent the re-do procedures with 1.6 ± 0.7 and 2.0 ± 1.0 procedures (p = 0.02). In LINE-group, mean number of reconnected PVs were 2.8 ± 0.9 PVs/patient, and reconnection of roof and MI lines was observed in 9 (38%) and 13 (54%) patients during the second procedure. All reconnected PVs were successfully re-isolated and bidirectional block was established across all lines. EGM-guided ablation was added in 7 (29%) patients. In EGM-group, all reconnected PVs (2.9 ± 0.9 PVs/patient) were also successfully re-isolated, and additional roof and MI lines were created in 24 (71%) and 27 (79%) patients during the second procedure. Non-PV foci, (including SVC in a subset) were detected in 21 (36%) patients. The incidence of non-PV triggers was comparable between the two groups (LINE-group vs. EGM-group: 8 [36%] vs. 13 [38%], p = 0.92). However, multiple ATs were more frequently induced during the second procedure in EGM-group (LINE-group vs. EGM-group: 7 [21%] vs. 0 [0%], p = 0.03). After taking multiple procedures into account (1.8 ± 0.9 procedures), 66/104 (63%) maintained SR with and without AADs in this population. AAs-free survival remained significantly higher in LINE-group as compared to EGM-group after the last procedure (LINE-group vs. EGM-group: year 1: 96% vs. 88%; year 3: 92% vs. 71%; year 5: 83% vs. 62%; year 8: logrank test: 88% vs. 65%, p = 0.045, Fig. 1B). Mean follow-up duration after the last procedure in patients who underwent redo-procedures was 59 ± 33 months. Overall 75/104 (72%) patients were AAs-free at 8 years after the last CA. The proportion of AAs-free patients on AAD therapy were comparable between the two groups (LINE-group vs EGM-group; 8/43 [19%] vs 8/32 [25%], p = 0.57). In addition, fewer CA procedures were required to maintain SR in the LINE-group as compared to EGM-group (1.6 ± 0.7 vs. 2.0 ± 1.0, p = 0.02).

### Predictors of AAs recurrence after the initial CA

In univariable analysis, longer AF duration, EGM-guided ablation and AF at the beginning of the index CA procedure were identified as predictors of AAs recurrence after the index ablation. Of note, incomplete line block and non-termination during EGM-guide ablation did not predict AAs recurrence. In multivariable analysis, longer AF duration and EGM-guided ablation remained as independent predictors of AAs recurrence (OR: 1.01, 95% CI 1.00–1.03, p = 0.015, OR: 2.45, 95% CI 1.47–4.09, p = 0.0007, respectively, Table 3).

**Table 3:** Predictors of AAs recurrence after the initial CA.

	Univariable analysis			Multivariable analysis		
	OR	95% CI	p-value	OR	95% CI	p-value
Age, years old	0.71	0.97–1.02	0.71			
Minimum continuous AF duration, month	1.01	1.00–1.03	**0.033**	1.01	1.00–1.03	**0.015**
Unknown time of AF onset	0.96	0.55–1.66	0.87			
BMI, kg/m^2^	0.99	0.93–1.06	0.82			
Hypertension	0.96	0.60–1.56	0.88			
CHA2DS2-VASc score	0.27	0.61–1.15	0.26			
LAD, mm	1.01	0.96–1.06	0.82			
LVEF, %	1.00	0.97–1.03	0.95			
BNP, pg/ml	1.00	0.99–1.00	0.95			
Ccr, ml/min	0.99	0.98–1.00	0.16			
Start in AF rhythm	2.24	1.03–4.90	**0.044**	1.78	0.80–3.94	0.16
EGM-guided ablation	2.62	1.59–4.34	**0.0002**	2.45	1.47–4.09	**0.0007**
Non-PV foci including SVC	1.46	0.73–2.95	0.29			

Significant values are in bold.

*AF* atrial fibrillation, *BMI* body mass index, *BNP* brain natriuretic peptide, *Ccr* creatinine crealance, *LAD* left atrial diameter, *LVEF* left ventricular ejection fraction, *OR* odds ratio, *SVC* superior vena cava.

## Discussion

The present study is the first report to directly compare the very long-term clinical outcome, spanning 8-years, between linear ablation (roof and MI lines) and EGM-guide ablation in addition to PVI for PsAF. Using a propensity score-matched analysis, we demonstrated superiority of linear ablation to EGM-guided ablation following single and multiple procedures. In addition to continuous AF duration, EGM-guided ablation was an independent predictor of arrhythmia recurrence after the index ablation procedure.

### Linear ablation for AF

Multiple previous studies have reported that linear ablation for PsAF is associated with favorable medium term outcomes, with success rates of 69–88% at 1–1.5 years after CA^6,18,19^. Gaita et al. reported that PVI plus linear ablation is superior to PVI alone in maintaining SR during a maximum follow-up period of 3 years^20^. However, the superiority of additional linear ablation has not been consistently demonstrated compared with PVI alone^9,10,21^. The concept of linear ablation was derived from the evidence of high success rates of the surgical MAZE procedure^22^. Roof and MI lines have a potential benefit for alteration of AF wavelet propagation and elimination of spectral components by complete bidirectional block across linear lesions. In addition, these linear lesions may lead to reduction of excitable LA myocardial mass, attenuation of vagal innervation^23^, and elimination of non-PV foci, especially those relating to the ligament of Marshall^24,25^. Although the endpoint of linear ablation is clear, establishing linear block, especially during MI line ablation, maybe challenging. Incomplete linear ablation has been associated with the occurrence of macro-reentrant AT^26,27^, and AF recurrences^28,29^. In the present study, although acute success rates of linear ablation were relatively high (94% in roof line, 81% in MI line), roof and MI lines were reconnected in 38% and 54% of patients at re-do procedures. Nevertheless, the incidence of linear gap-related AT was observed in only 13%, which was statistically similar with EGM-guided ablation group. These results may suggest that complete linear block is important for the modification of AF substrate, rather than for avoiding occurrence of macro-reentrant AT. It was possible to create durable linear lesions by using steerable sheaths in our study compared with other previous studies, which may have led to better clinical outcomes.

### EGM (CFAE)-guided ablation for AF

The original CFAE ablation study, reported by Nademanee et al., demonstrated high acute AF termination rates of 82% and arrhythmia-free survival in three quarters of patients at one-year follow-up^7^. Haissaguerre et al. subsequently proposed EGM-guided ablation targeting fractionated, rapid and gradient signals in addition to linear ablation, an approach associated with a high acute AF termination rates^8^. The concept of EGM or CFAE-guided ablation involves targeting AF driver sources represented by pivot points of wavelets, continuous re-entry of fibrillation waves with relatively short cycle lengths, with heterogenous temporal and spacial distribution^30^. Of note however, identification of CFAE signals is quite subjective (e.g. definition of fractionated electrograms) and therefore this strategy is not consistent and also not formally established, even with the use of automatic analysis algorithms incorporated into 3D-EAM systems^21,31^. A recent large meta-analysis did not demonstrate an incremental beneficial effect of CFAE ablation in addition to PVI compared with PVI alone in PsAF patients^9^. It is possible that in addition to critical AF drivers CFAEs also represent passive activation, signal artifact, double potentials, slow conduction, and wavebreak^15,32^. In the present study, EGM-guided ablation, using the Bordeaux approach^8^, was performed by visual and manual inspection by sufficiently experienced operators. However, methodological differences might be associated with lower AF termination rates (40%) and poor clinical outcomes (AF/AT-free survival of 38%/1.5 years). These limitations are important to consider when comparing outcomes of CFAE ablation is the inter-operator variability CFAE identification.

### Direct comparison of clinical outcome between linear and EGM (CFAE)-guided ablation

Although previous meta-analyses comparing different strategies for PsAF ablation in the different cohorts reported non-superiority of additional substrate modification beyond PVI, the studies were associated with heterogenous patient populations and shorter follow-up periods^9,21^. Only two previous studies have prospectively compared linear ablation and CFAE ablation in PsAF patients. In contrast to our results, both studies reported no significant differences in arrhythmia-free survival between the two groups during medium-term follow-up^11,12^. While Estner et al. reported similar outcomes between linear ablation and CFAE ablation at one year, the success rates were lower than those in the present study (linear vs. CFAE: 37% vs. 39%) despite high success rates of linear block (100%) and AF termination (82%) during CFAE ablation. Of note, their ablation strategy in the linear ablation group included roof and anterior lines in only two thirds of patients, and mean LA size was larger (47–49 mm)^12^. These results may suggest that the effect of linear ablation, especially anterior line ablation, is limited in advanced PsAF. In the STAR-AFII trial, there was no difference in AF/AT-free survival between linear ablation (roof and MI lines) and CFAE ablation during 1.5 years of follow-up (41% vs. 37%). Regarding procedural results, AF terminated in 45% by CFAE ablation, and successful linear ablation was achieved in 93% and 75% of roof and MI lines, respectively^11^. There are a number of potential explanations for the inconsistent results between our study and STAR AF II. Firstly, the acute success rate of MI line block was lower than our study (81%). Secondly, our ablation protocol included ablation for non-PV foci. Thirdly, we confirmed elimination of dormant conduction post-PVI with isoproterenol and adenosine after 30 min waiting in all patients. Fourthly, a steerable sheath was used for linear ablation, which may have contributed to the higher success rates in the linear ablation group^33^. Overall, our study showed consistent efficacy of linear ablation in PsAF patients during very long-term follow-up (> 8 years) after the initial and multiple catheter ablation procedures with fewer procedures compared to the EGM-guided ablation cohort. Multiple ATs were more frequently induced at the re-do procedure in the EGM-guided ablation group. These findings may suggest that some of the critical CFAE areas were not targeted during the index ablation^15,32^, and potential incompletely ablated areas may create a substrate for iatrogenic arrhythmias^34^.

### Limitations

There are a number of potential limitations associated with the present study. First, the study has the inherent limitations of a non-randomized retrospective study from a single-center. While we used propensity score-matched analysis based on multiple baseline characteristics for statistical adjustment, selection bias was not fully eliminated. Second, we did not use force sensing catheters or high-power short duration ablation, which may influence generalizability to contemporary practice. Third, our cohort was younger with a dominance of male patients and therefore, the results may not be generalizable to older and female patients. Fourth, while it would have been of value to compare outcomes of EGM-guided and EGM-guided + linear ablation, the number of patients with EGM-guided + linear ablation was too small (n = 5). Fifth, we used LA dimension instead of LA volume, which is a less accurate reflection of LA size. Finally, shorter periods of ambulatory monitoring may have failed to detect AF episodes, as only 10% of patients had more prolonged (5-day) ambulatory monitoring. Furthermore, restricting ambulatory monitoring to symptomatic patients could potentially miss asymptomatic arrhythmia recurrences.

## Conclusions

Our single center retrospective analysis demonstrated that linear ablation might be a more effective complementary technique to PVI than EGM-guided ablation with more favorable outcomes during very long-term follow-up period.

## Methods

### Study population

A total of 325 PsAF patients who underwent a first-time CA in our institution between September 2008 and July 2015 were retrospectively included. In this population, PVI plus linear ablation or EGM-guided ablation was performed in 230 and 82 patients, respectively. Following propensity-matched score analysis, 104 patients (LINE-group: 52, EGM-group: 52) were included in the analysis. PsAF was defined as AF lasting more than 7 days with or without anti-arrhythmic drugs, and long-standing persistent AF was defined as continuous AF for more than 12 months^13^. AF duration was defined as the duration from the date when the AF was detected on 12-lead electrocardiogram (ECG) to either the date of AF termination by medical/electrical cardioversion, or the date of the initial CA procedure (if AF persisted despite medical/electrical cardioversion).

Clinical investigations were conducted in accordance with the principles expressed in the Declaration of Helsinki. All data were compliant with the International Conference on Harmonization guidelines. The study was approved by the ethics committee of The Jikei University School of Medicine for Biomedical Research. All methods were carried out in accordance with relevant guideline and regulations. All patients provided their written informed consent.

### Catheter ablation (CA)

Pre-procedure preparation and techniques for PsAF ablation have been described previously^14,15^. In brief, all patients received oral anticoagulation at least 4 weeks prior to the ablation procedure. Anti-arrhythmic drugs were discontinued for at least five half-lives prior to ablation. After exclusion of LA thrombus using trans-esophageal echocardiography, CA was performed under mild deep sedation. Continuous and bolus heparin administered with a target activated clotting time of 300–400 s.

The initial ablation step involved segmental antral PVI (in AF or SR) with the guidance of a large circular mapping catheter (Lasso 20–30 mm; Biosense-Webster Inc, Diamond Bar, CA, USA). Ablation was performed using a non-contact force sensing open-irrigated 3.5 mm-tip ablation catheter (Cool Path™ Duo, FlexAbility™; St jude Medical or ThermoCool/SF; Biosense Webster) with a power limit of 25–35 W. The procedural endpoint of PVI was bidirectional electrical conduction block between the LA and PVs^14^. Following PVI, linear ablation or EGM-guided ablation was performed.

In LINE-group, following roof line ablation (connecting the superior PVs), MI line ablation was performed with the endpoint of a bidirectional electrical conduction block across the lines^16,17^. MI line ablation was performed from the mitral annulus to the lower pole of the left inferior PV along the shortest line located (3–4 o’clock in the left-anterior oblique view). Epicardial ablation within the CS was subsequently added in cases with incomplete block from endocardial ablation. Ablation setting during linear ablation was as follows: a power output of 30–40 W on the roof and endocardial MI and 20–25 W on the epicardium within the CS.

In the EGM-group, ablation was performed at all sites displaying any of the following EGM features: (1) continuous electrical activity, (2) rapid activity with cycle length (CL) shorter than the mean LA appendage AF CL, (3) complex and fractionated electrograms, and (4) a gradient of activation (a temporal gradient of at least 70 ms between the distal and proximal bipoles on the map electrode, potentially representing a local circuit) with the endpoint of AF termination^8^. The power output during EGM-guided ablation was 30 W except for the posterior wall adjacent to the esophagus (20 W with ablation termination upon abolition of the local specific electrograms). Electrical cardioversion was performed at the end of the procedure where elimination of targeting signals in both right and left atrium failed to terminate AF.

In all cases, the presence/absence of dormant conduction was tested by administration of isoproterenol (4 µg) and adenosine triphosphate (10–20 mg) in each PV after a minimum waiting period of 30 min after PVI. Dormant conduction was eliminated by additional RF applications. In cases with evidence of non-PV foci, the ablation strategy involved targeting non-PV foci. SVC isolation was performed if arrhythmogenicity was detected in the SVC. Sustained AT occurring during the procedure was also mapped and ablated. Carvotricuspid isthmus linear ablation was routinely performed in all patients regardless of the presence or absence of documented typical atrial flutter. 3D-electroanatomical mapping (EAM) systems (CARTO3, Biosense-Webster Inc. or Navx, St Jude Medical, Inc, Minneapolis, MN, USA) were used during CA.

In the case of re-do procedures, the ablation strategy involved re-isolation of pulmonary veins (where indicated), and/or additional EGM-guide ablation, and/or linear ablation (roof and MI lines where necessary) with operator’s discretion. Non-PV foci were targeted during the re-do procedure in the same way as the index procedure (following administration of isoproterenol [4 µg] and adenosine triphosphate [10–20 mg]). In case with AT recurrence, the arrhythmia was targeted with AT non-inducibility as the endpoint (no AT inducible with burst pacing up to 200 ms).

### Acute outcomes

Acute procedure related complications (cardiac tamponade, air embolism and major bleeding) were documented. Very early recurrences were defined as recurrences occurring within three days of the procedure.

### Follow-up

Patients were followed-up with 12-lead ECG and 24-h Holter ECG at 1, 3, 6 and 12 months and subsequently every 6–12 months. Amongst symptomatic patients in whom 24-h monitors failed to document arrhythmia, a 5-day event recorder or portable ECG recorder (HeartScan, Omron Healthcare, Kyoto, Japan) was used. The primary end point was a recurrence of AAs, which included AF or AT. AA recurrence was defined as the presence of AF/AT lasting > 30 s after the blanking period of 3 months. AADs were discontinued 3–12 months post ablation in cases without AAs recurrence at the treating physician’s discretion.

### Statistical analyses

Due to differences in baseline characteristics between patients in the linear ablation versus EGM-guided ablation groups, propensity-score matching was used to identify a subsets of patients with similar baseline characteristics. Patients were matched in a 1:1 ratio based propensity scores. Propensity scores were calculated for each patient using multivariable logistic regression with following 14 covariates: age, gender, body mass index, AF duration, long-standing AF, asymptomatic AF, the number of failed AADs, structure heart disease, brain natriuretic peptide (BNP), estimated glomerular filtration rate, hypertension, diabetes mellitus, congestive heart failure, CHA2DS2-VASc score, LAD and LVEF, using a caliper width equal to 0.2 of the standard deviation of the logit of the propensity score. Quantitative data were expressed as the mean ± standard deviation. Comparisons between groups were performed using an unpaired Student’s *t*-test or Wilcoxon’s rank-sum test (based on the distribution of the values). Comparisons of means from the same individual were performed using a paired Student’s *t*-test. Categorical data were compared by χ^2^ test. Kaplan–Meier curves were used to analyze freedom from AAs, and groups were compared using the log-rank test. The multivariable analysis was described by Cox regression for the assessment of the predictors of AAs recurrence after the initial CA procedure. All tests were 2-tailed, and P values of < 0.05 were considered to indicate statistical significance. All of the statistical analyses were performed using the MedCalc software package, version 11.2 (MedCalc Software, Mariakerke, Belgium).
